# Machine learning in perioperative medicine: a systematic review

**DOI:** 10.1186/s44158-022-00033-y

**Published:** 2022-01-15

**Authors:** Valentina Bellini, Marina Valente, Giorgia Bertorelli, Barbara Pifferi, Michelangelo Craca, Monica Mordonini, Gianfranco Lombardo, Eleonora Bottani, Paolo Del Rio, Elena Bignami

**Affiliations:** 1 Anesthesiology, Critical Care and Pain Medicine Division, Department of Medicine and Surgery, University of Parma, Viale Gramsci 14, 43126 Parma, Italy; 2 General Surgery Unit, Department of Medicine and Surgery, University of Parma, Viale Gramsci 14, 43126 Parma, Italy; 3 Department of Engineering and Architecture, University of Parma, Viale G.P.Usberti 181/A, 43124 Parma, Italy

**Keywords:** Risk prediction, Surgery, Machine learning, ICU, Anesthesia, Perioperative

## Abstract

**Background:**

Risk stratification plays a central role in anesthetic evaluation. The use of Big Data and machine learning (ML) offers considerable advantages for collection and evaluation of large amounts of complex health-care data. We conducted a systematic review to understand the role of ML in the development of predictive post-surgical outcome models and risk stratification.

**Methods:**

Following the Preferred Reporting Items for Systematic Reviews and Meta-analyses (PRISMA) guidelines, we selected the period of the research for studies from 1 January 2015 up to 30 March 2021. A systematic search in Scopus, CINAHL, the Cochrane Library, PubMed, and MeSH databases was performed; the strings of research included different combinations of keywords: “risk prediction,” “surgery,” “machine learning,” “intensive care unit (ICU),” and “anesthesia” “perioperative.” We identified 36 eligible studies. This study evaluates the quality of reporting of prediction models using the Transparent Reporting of a Multivariable Prediction Model for Individual Prognosis or Diagnosis (TRIPOD) checklist.

**Results:**

The most considered outcomes were mortality risk, systemic complications (pulmonary, cardiovascular, acute kidney injury (AKI), etc.), ICU admission, anesthesiologic risk and prolonged length of hospital stay. Not all the study completely followed the TRIPOD checklist, but the quality was overall acceptable with 75% of studies (Rev #2, comm #minor issue) showing an adherence rate to TRIPOD more than 60%. The most frequently used algorithms were gradient boosting (*n* = 13), random forest (*n* = 10), logistic regression (LR; *n* = 7), artificial neural networks (ANNs; *n* = 6), and support vector machines (SVM; *n* = 6). Models with best performance were random forest and gradient boosting, with AUC > 0.90.

**Conclusions:**

The application of ML in medicine appears to have a great potential. From our analysis, depending on the input features considered and on the specific prediction task, ML algorithms seem effective in outcomes prediction more accurately than validated prognostic scores and traditional statistics. Thus, our review encourages the healthcare domain and artificial intelligence (AI) developers to adopt an interdisciplinary and systemic approach to evaluate the overall impact of AI on perioperative risk assessment and on further health care settings as well.

## Background

Risk stratification is a central part of the anesthetic evaluation. In fact, through the identification of high-risk patients, it is possible to conduct a specific risk/benefit analysis, to reduce the risk of unexpected complications, to achieve a targeted perioperative optimization, to carefully plan the anesthesiologic management, and to provide an accurate and precise informed consent [1–3].

Over time, several scores have been published, from the most generic, like the American Society of Anesthesiologists Physical Status (ASA-PS) [4], to the most specific ones, as the European system for cardiac operative risk evaluation (EuroSCORE) [5] or the General Surgery Acute Kidney Injury Risk Index Classification System [6]. Unfortunately, these scores have some limits, mainly due to the lack of tailored predictions.

In the last decade, the interest about artificial intelligence (AI), including machine learning (ML) methods, have seen an exponential increase [2]. Considered an extension of traditional statistics, AI differs from standard approaches for its ability to learn from examples and mistakes, to improve continuously with the introduction of new data, and to create a model for individualized patient care [7].

Thanks to the growing informatization of health systems, large amounts of data have become available. The implementation of new technologies and the development of prediction algorithms paved the way for novel possibilities to exploit these huge data collections. Among the several branches of healthcare in which ML aroused enthusiasm, its application in perioperative medicine is showing promising results. In fact, in consideration of its specific characteristics, this analytical technique is suitable for the creation of predictive models, specifically concerning the optimization of resources and the development of warning score systems [8, 9]. The application of these algorithms allows early detection and prediction of acute critical illness, facilitating the management of high-risk patients [10].

More recently, COVID-19 pandemic lighted on the importance of AI-based models for the fast development of algorithms that could integrate readily available data, helping the hospital systems and the clinicians in optimal patient care [11].

The use of ML techniques for the creation of predictive models of perioperative complications is in continuous expansion.

The aim of our review is to clarify the role of ML in perioperative settings, evaluating currently available predictive outcome models, the types of ML algorithms used more frequently, and their proved efficacy.

## Methods

### Literature search

This systematic review was conducted according to Preferred Reporting Items for Systematic Reviews and Meta-analyses (PRISMA) guidelines (http://prisma-statement.org/documents/PRISMA_2020_checklist.pdf).

The authors performed a systematic literature search of Scopus, CINAHL, the Cochrane Library, PubMed, MeSH, MEDLINE, and Embase, from 1 January 2015 to 30 March 2021, using different combinations of the following terms: “risk prediction,” “surgery,” “machine learning,” “ICU,” “anesthesia,” and “perioperative.”

Specifically, ((((((("risk prediction"[All Fields]) AND ("surgery"[All Fields])) AND ("machine learning"[All Fields])) OR (risk prediction)) ) AND (machine learning)) AND (ICU)) OR (risk prediction)) AND (machine learning)) AND (anesthesia); ((((((((("risk prediction"[All Fields]) AND ("surgery"[All Fields])) AND ("machine learning"[All Fields])) OR (risk prediction)) ) AND (machine learning)) AND (ICU)) OR (risk prediction)) AND (machine learning)) AND (anesthesia) OR (((((((("risk"[All Fields]) AND ("surgery"[All Fields])) AND ("machine learning"[All Fields])) OR (risk)) ) AND (machine learning)) AND (ICU)) OR (risk)) AND (machine learning)) AND (anesthesia); ((postoperative) AND machine learning) AND (intensive care admission).

In the last 10 years, there was an exponential increase in literature concerning the application of AI in medicine. Therefore, we decided to perform the search in this time frame to include more homogeneous and easily comparable studies. We included studies if they evaluated ML models in surgical settings for the prediction of perioperative risk. Both prospective and retrospective studies were eligible for inclusion. The following types of study were excluded: papers published prior to 2015, papers concerning outpatient settings, animal studies, pediatric population, and studies written in languages other than English. Furthermore, primary study evaluating strictly surgical outcomes, and systematic reviews were considered uneligible.

### Data extraction and quality assessment

The primary aim of our study was to assess the main perioperative outcomes in which ML methods are used, and their efficacy among different algorithms.

Two reviewers independently screened the selected articles, and a third reviewer resolved any discrepancies.

To assess the reporting quality of all included studies, we used the Transparent Reporting of a multivariable prediction model for Individual Prognosis or Diagnosis (TRIPOD) checklist [12]. In fact, it provides guidance for extracting relevant information and calculating summary scores to determine adherence of primary prediction model to the TRIPOD.

Two independent reviewers assessed for each selected study the compliance with the items described in the checklist. Moreover, to facilitate data extraction and scoring, the studies were analyzed according to the study design, predictor selection, outcome assessment, applied model, and its validation. The checklist includes 22 main items, of which ten are divided in sub items, all with four potential answer options: “yes,” “not,” “referenced,” “not applicable.” After adequately fulfilling each item of the checklist, the adherence to the TRIPOD is automatically calculated. We established different levels of adherence to TRIPOD, setting a scale from 0 to 100%, assuming that a research was more accurate with higher adherence to tripod checklist.

## Results

**Fig. 1:**
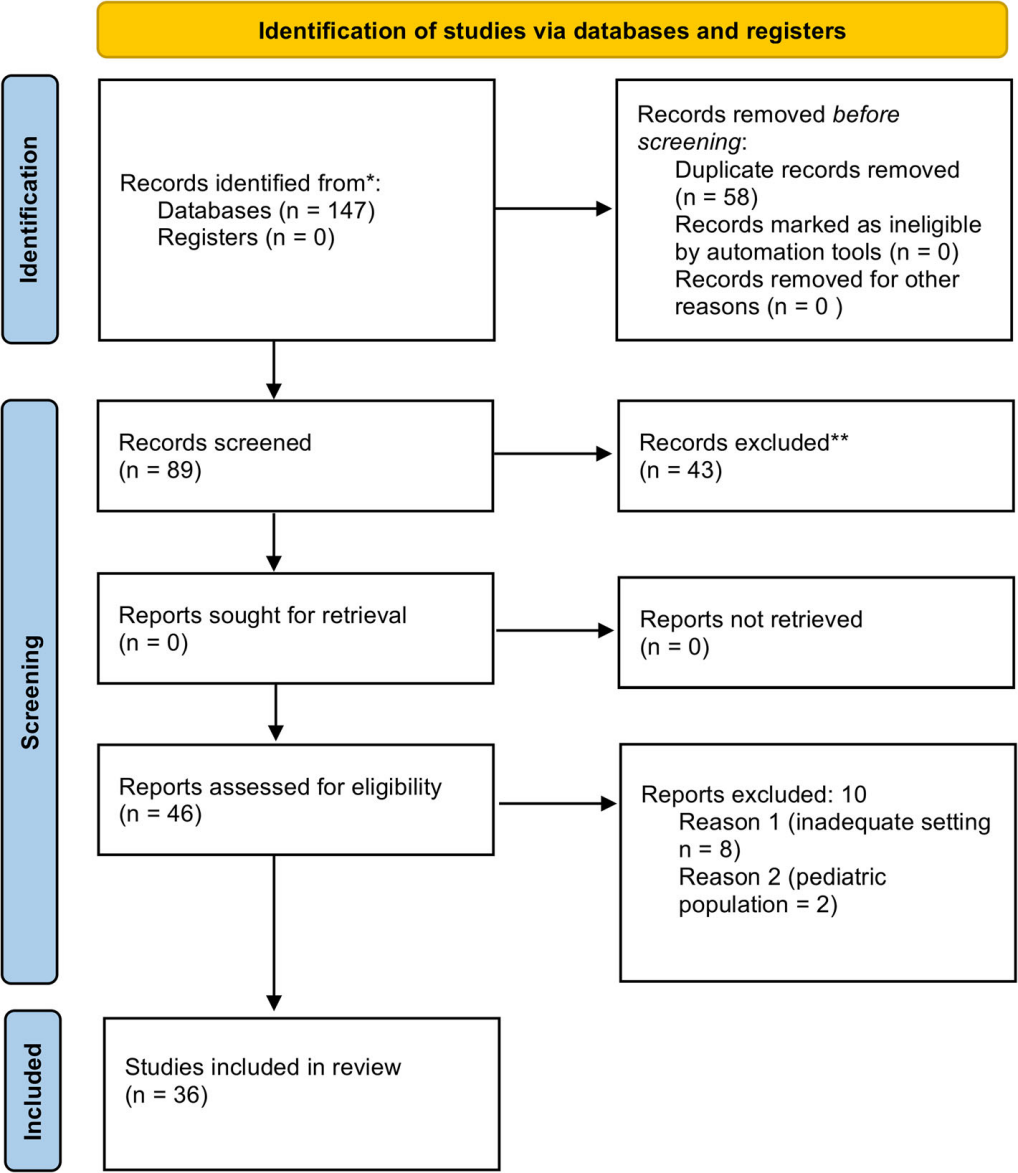
Preferred Reporting Items for Systematic Reviews and Meta-Analysis (PRISMA flow chart) illustrating the process of selecting eligible publications for inclusion in the systematic review

One hundred forty-seven papers were identified through database searching. After the removal of the duplicates, 89 articles were screened, and 43 were found to be ineligible after reading the abstracts. Out of the 46 full text reviewed articles, 10 were excluded because of inadequate clinical setting or because concerning pediatric population. Finally, 36 articles were included for the review (Fig. 1).

Outlines all characteristics of the final selected articles (Table 1) [13–48], including the design, cohort, and objective of each study, as well as the ML methods used and the best performance.

**Table 1:** Overview of papers included in our analysis

Author, years	Study design	Objective	Final cohort	Outcomes	Type of ML	Prediction performance	Comparator/control
Lundberg SM, 2019	Retrospective/observational single center	Development and testing of a ML-based system that predicts the risk of hypoxemia during general anesthesia	48,069	Hypoxemia	GB	AUC 0.92	ML-based system was compared to anesthesiologists’ predictions
Kendale S, 2018 [13]	Retrospective/observational single center	Prediction of the risk of post-induction hypotension using ML methods	13,323	Cardiovascular complications	RF, SVM, GB, BN, LR-EN, regularization, K nearest; linear discrimination analysis; neural nets	AUC GB 0.74 (95% CI, 0.72 to 0.77). RF 0.74 (95% CI, 0.73 to 0.75)	Different ML algorithms were trained to obtain the model with the best performance
Fernandes MPB, 2021 [14]	Retrospective/observational single center	ML models used to predict postoperative mortality rarely include intraoperative factors.	5015	Mortality	logistic regression, RF neural networks, SVM and extreme gradient boosting (XGB).	XGB predicted mortality confidence interval (CI): 0.88 (0.83–0.94)	Different ML algorithms were trained to obtain the model with the best performance
Cherifa M, 2020 [15]	Retrospective/observational single center	Prediction of acute hypotensive episode	1151	Cardiovascular complications	Super Learner (SL) algorithm	SL AUROC 0.890	Different ML algorithms were trained to obtain the model with the best performance
Flechet M, 2019 [16]	Prospective/observational single center	Compare diagnostic performances of ML models and physicians in predicting AKI-23 in the 7 days following ICU admission	252	Acute kidney injury	ML based AKI predictor	AUROC 0.80	Physicians’ predictions were compared against the AKI predictor model
Kang AR, 2020 [17]	Retrospective/observational single center	Prediction of hypotension during anesthesia induction	222	Cardiovascular complications	Naïve Bayes, logistic regression, RF, ANN	RF best performance AUC 0.842	Different ML algorithms were trained to obtain the model with the best performance
Meiring C, 2018 [18]	Retrospective/observational multicentric	Identification of risk factors for admission in ER/ICU for spine patients	11150	ER/ ICU admission	RF, SVM, GB, DECISION TREE, DEEP LEARNING, NNC, Single layer averaged neural network	RF AUC 0.859, NNC AUC0.864; SVM AUC 0.867; adaboost AUC 0.868; deep learning AUC 0.883	Logistic regression against physiological data alone outperformed APACHE-II (current risk stratification tools)
Nudel J, 2021 [17]	Retrospective/observational multicentric	Comparison of two ML strategies with conventional statistical models in prediction of surgical complication	43,6807	Surgical complications, VTE	GB, ANN	ANN, and XGB, LR achieved similar AUCs 0.65, 0.67 and 0.64	Different ML algorithms were trained to obtain the model with the best performance
Lee Hc, 2018 [19]	Retrospective/observational single center	Comparison of ML method with logistic regression analysis to predict AKI after cardiac surgery	2010	AKI, mortality	RF, SVM, GB, DECISION TREE, DEEP LEARNING, NNC	Best GB AUC 0.78	The performance of ML approaches was compared with that of LR analysis
Bai P, 2020 [20]	Retrospective/observational multicentric	Identification of risk factors of early cerebral infarction and myocardial infarction after CEA with ML method	443	Cardiovascular complications	linear SVM, decision tree,RF,ANN, quadratic discriminant analysis, and XGBoost	XGBoost had the highest accuracy	Not applicable
Tan HS, 2021 [2021]	Retrospective study single center	Use of ML to identify predictive factors for inadequate labor anesthesia	20,716	Pain prevention	RF, XGBoost and logistic regression models	All three models performed similarly, with AUC 0.763–0.772	The performance of ML was compared with regression techniques
Solomon SC, 2020 [21]	Retrospective and prognostic single center	Prediction of intraoperative bradicardia	62,182	Cardiovascular complications	Gradient Boosting Machine (GBM)	AUC of 0.81–0,89	The performance of ML was compared with regression techniques
Ko S, 2020 [22]	Retrospective and multicentric	Prediction of postoperative AKI after total knee arthroplasty.	5757	AKI	Gradient Boosting Machine (GBM)	AUC of 0,78	Not applicable
Lu Y, 2020 [23]	Retrospective single center	Develop ML algorithm for identification of patients requiring admission following elective anterior cruciate ligament (ACL) reconstruction.	4709	Length of stay	RF, XGBoost, LDA, AdaBoost	The ensemble model achieved the best AUC 0.76	Not applicable
Maheshwari K, 2020 [24]	Observational single center	Using ML to predict intraoperative hypotension	305	Cardiovascular complications	Hypotension Prediction Index	95% confidence interval	Not applicable
Hill BL, 2019 [25]	Retrospective/observational single center	Develop a model that estimates in-hospital mortality at the end of surgery to quantify the change in risk during the perioperative period.	53,097	Mortality	Logistic regression, Elastic Net24 logistic regression, RF, GB.	Best RF 0.932	Comparison of ML methods with the perioperative score (as ASA physical status score)
Suhre W,2020 [26]	Retrospective multicentric	Correlation between chronic cannabis use and the risk of postoperative nausea and vomiting (PONV).	16,245	PONV	Bayesian additive regression trees (BART)	90% CI 0.98–1.33	Not applicable
Lee HC, 2018 [27]	Retrospective/observational single center	Comparison of ML method with logistic regression analysis to predict AKI after liver transplantation	1211	AKI, mortality	RF, SVM, GB, Decision tree, Neural network Classifier, BN, LR-EN, multilayer perceptron	Best GB AUC 0.90	The performance of ML approaches was compared with that of LR analysis
Barry GS, 2021 [28]	Retrospective cohort study	Investigate the incidence and factors associated with rebound pain in patients who received a PNB for ambulatory surgery.	482	Pain control	Logistic model tree attribute-selected classifier	ROC curve of 0.609	Not applicable
Gabriel RA, 2019 [29]	Retrospective/observational single center	Develop a predictive model for determining LOS.	1018	LOS	Ridge regression, Lasso, RF	ridge regression 0.761, Lasso 0.752, RF 0.731	Predictive models using ML techniques were compared to model performances
Li H, 2020 [30]	Retrospective/observational single center	Development of a predictive model for LOS after total knee arthroplasty	1826	LOS	GB	AUC 0.738.	Logistic regression and ML model were compared
Jungquist CR, 2019 [31]	Retrospective/observational single center	Early detection of respiratory depression using ML models	60	Postoperative respiratory complications	SVM	Accuracy of 80%	Not applicable
Nguyen M, 2020 [32]	Multicentric randomized	Using ML techniques and causal inference methods to detect the dynamic relationship between transfusion ratios and outcomes in trauma patients	680	Mortality and hemorrhagic complications	Statistical programming language R	Mortality at AUC 0.89, hemorrhagic complications 1.07	ML techniques were used to augment the intent-to-treat analysis of PROPPR
Tourani R,2019 [33]	Retrospective multicentric	In the context of perioperative decision support, understand if the use of intraoperative data improve the performance of 30-day postoperative risk models	38,045 + 9,044	Sepsis, septic shock, UTI, PNA, surgical infections	Logistic regression models.	AUC between 0.66 and 0.82	Not applicable
Cartailler J,2019 [34]	Clinical trial single center	Use of EEG-patterns to anticipate excessive deep sedation	80	Neurological complications	RF	AUC of 0.93	Not applicable
Wong WEJ, 2021 [35]	Retrospective/observational single center	Prediction of AKi in ICU	940	ICU AKI, hospital and 1 year mortality	Chi-square test, Fisher’s exact test,Mann-Whitney test, independent *t* test and the Kaplan-Meier curve.	AUROCs of the auxiliary models for ICU AKI were 0.7537, 0.7589, 0.7950, 0.7333 and 0.7654.	Not applicable
Lee CK, 2021 [36]	Retrospective/observational single center	Prediction of mortality in post-operative patients	59,985	Post-operative mortality	Generalized additive models with neural networks (GAM-NNs).	AUC 0.921	Model performance was compared to a standard LR model
Jeong YS, 2021 [37]	Retrospective/observational single center	To make a proper model for predicting postoperative major cardiac event (MACE) in ESRD patients undergoing general anesthesia.	3220	Cardiovascular complications, mortality	SVM, decision tree, RF, Gaussian naive Bayes (GNB), ANN, LR, XGBoost	RF AUC 0.797	Different ML algorithms were trained to obtain the model with the best performance
Filiberto AC, 2021 [38]	Retrospective/observational single center	Postoperative acute kidney injury using ML models	1531	AKI	RF	AUC 0.70	ML models using the perioperative data were compared to models using either preoperative data alone or the ASA physical status classification
Meyer A, 2018 [39]	Retrospective/observational single center	Use machine learning methods to predict severe complications during and after cardiothoracic surgery.	11,492	Postoperative bleeding, AKI, mortality	Deep learning model	AUC 0·09 for bleeding, of 0·18 for mortality, and of 0·25 for AKI	Deep learning methods were compare to established standard-of-care clinical reference tools
Chiew CJ, 2020 [40]	Retrospective/observational single center	Compare the performance of ML models against the traditionally (CARES) model and (ASA-PS) in the prediction of 30-day postsurgical mortality and ICU admission	90,785	Mortality, postoperative ICU admission	RF, GB, adaptive boosting, SVM	Best GB AUC 0.23 and for mortality and 0.38 ICU admission	The performance of ML models was compare against the traditionally Combined Assessment of Risk and Encountered in Surgery (CARES) model and the ASA physical status.
Bihorac A, 2019 [41]	Retrospective/observational single center	To calculate the risk for postoperative complications and death after surgery using ML	51,457	AKI, sepsis, VTE, ICU admission > 48 h, mechanical ventilation > 48 h, wound, neurologic and cardiovascular complications	MySurgeryRisk algorithm	AUC values ranging between 0.82 and 0.94	Not applicable
Yao RQ, 2020 [42]	Retrospective/observational single center	Develop a mathematical model for predicting the in-hospital mortality among patients with postoperative sepsis.	3713	Postoperative sepsi, mortality	Extreme gradient boosting (XGBoost) and stepwise logistic regression	Best XGBoost AUC 0.835	ML model was compare to the stepwise LR model.
Datta S, 2020 [43]	Retrospective/observational single center	Describe a model that predicts postoperative complications considering intraoperative events.	43,943	ICU LOS,prolonged mechanical ventilation, neurologic complications cardiovascular complications, AKI, VTE, wound complications, mortality	RF	AUC 0.21	ML models using preoperative and intraoperative data were compare to models using preoperative data alone
Brennan M, 2019 [44]	Prospective, non-randomized pilot study	Assess the usability and accuracy of the MySurgeryRisk algorithm for preoperative risk assessment	20	AKI, sepsis, VTE, ICU admission > 48 h, mechanical ventilation > 48 h, wound, neurologic and cardiovascular complications	MySurgeryRisk algorithm	MySurgeryRisk algorithm ranged between 0.73 and 0.85	Compare the accuracy of perioperative risk-assessment between physicians and MySurgeryRisk.
Houthooft R,2015 [45]	Retrospective/observational single center	develop model to determine patient survival and ICU length of stay (LOS) based on monitored ICU patient data.	14,480	LOS	ANN, k-nearest neighbors (k-NN), SVMs, classification trees (CART), RF, adaptive boosting (AdaBoost)	SVM AUC 0.77	Different ML algorithms were trained to obtain the model with the best performance

*AdaBoost* = adaptive boosting algorithms, *AKI* = acute kidney injury; *ANN* = artificial neural network models, *BART* = Bayesian additive regression trees, *BN* = Bayesian network, *GB* = gradient boosting, *ICU* = intensive care unit, *LDA* = linear discriminant classifier, *LOS* = length of stay, *LR-EN* = logistic regression with elastic net, *ML* = machine learning, *NNC* = neural network classifier, *PNA* = pneumonia, *PONV* = postoperative nausea and vomiting, *RF* = Random Forest, *SVM* = support vector machine, *UTI* = urinary tract infection, *VTE* = venous thromboembolism, *XGBoost* = extreme gradient boosting, *ASA* = American Society of Anesthesiologist

**Fig. 2:**
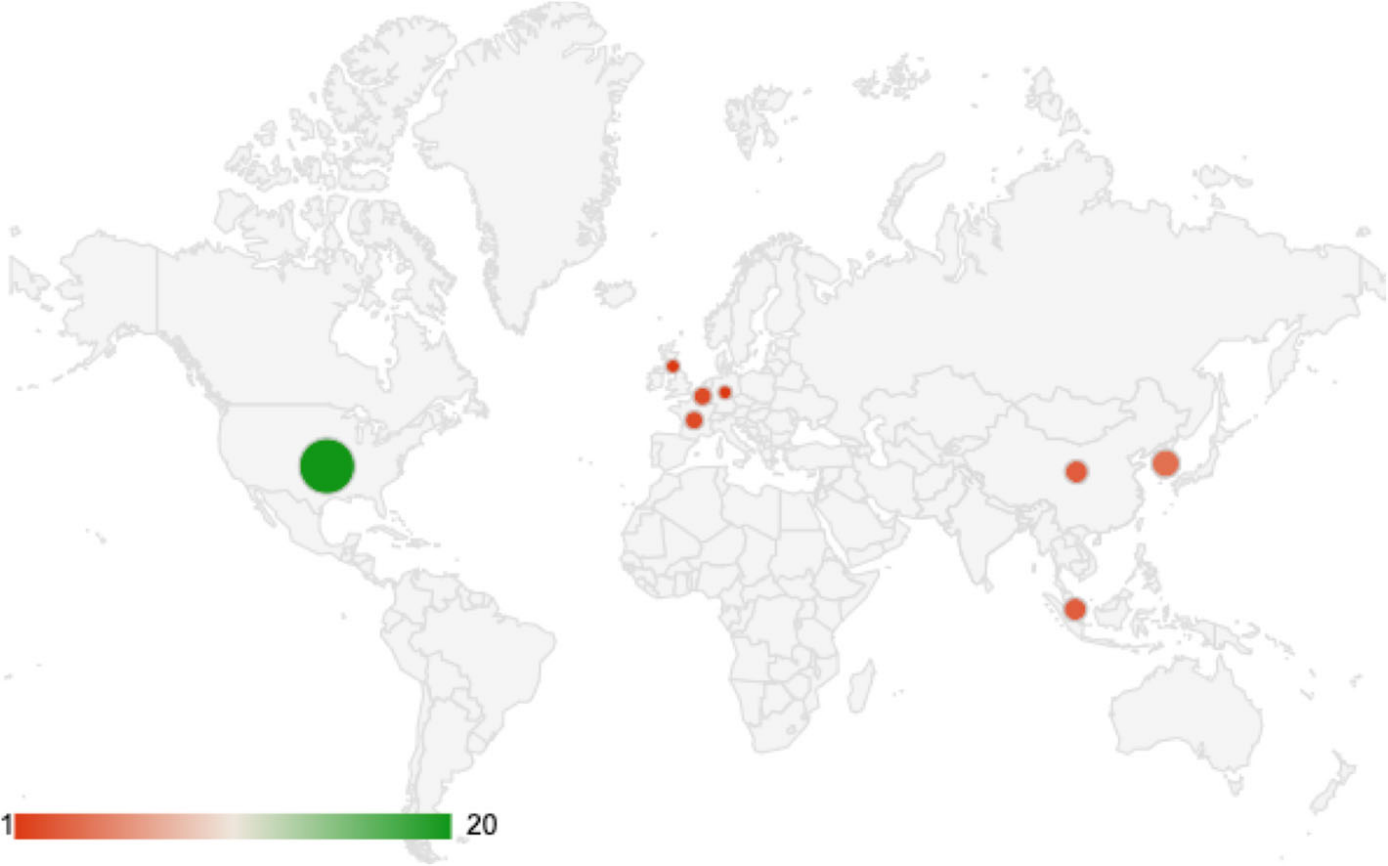
Geographical distribution of articles publications. The USA is the main country where publications came from, followed by China and Korea

Our analyses pointed out that more than 95% of included studies were published after 2018, and almost entirely performed in USA and Asia (Fig. 2).

**Fig. 3:**
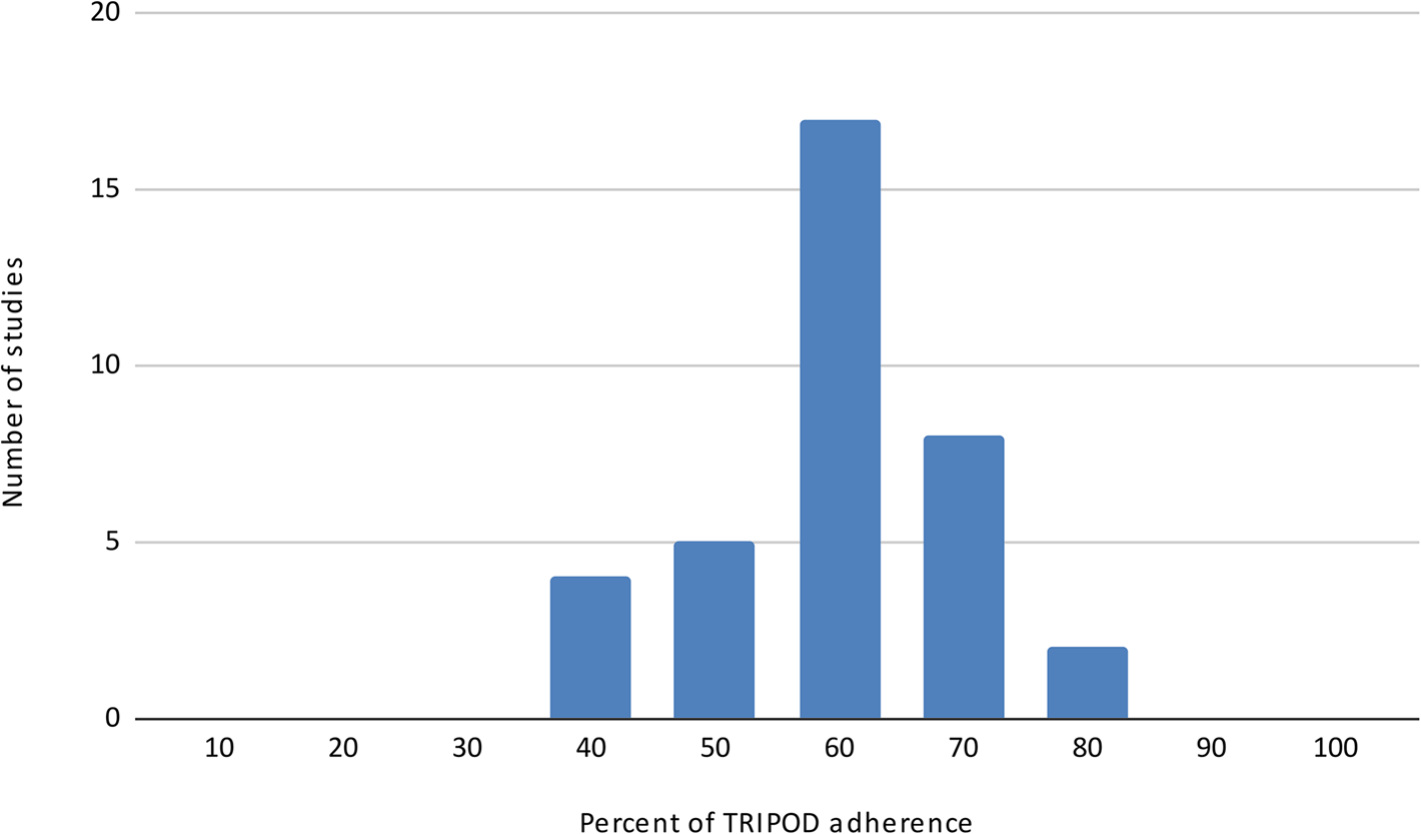
Frequency of adherence to TRIPOD checklist

The quality of the studies selected for the review was acceptable, with 75% of studied showing an adherence rate to TRIPOD more than 60% (Fig. 3). Specifically, in the first section of the checklist (Title and Abstract), a mean of 42% of studies adhere to tripod item. Concerning the methods section, all the articles defined the study design, or the source of data, while 53% of papers described the handling of missing data. In the results section, measures applied and models used were not always appropriated in the included studies, specifically 8% of papers presented the full prediction model and explained how to use it, while 19% of studies reported performance measures for the prediction model (Rev #2, comm #3).

Nearly all manuscripts discussed about the limitations of the study and gave an overall interpretation of results.

**Fig. 4:**
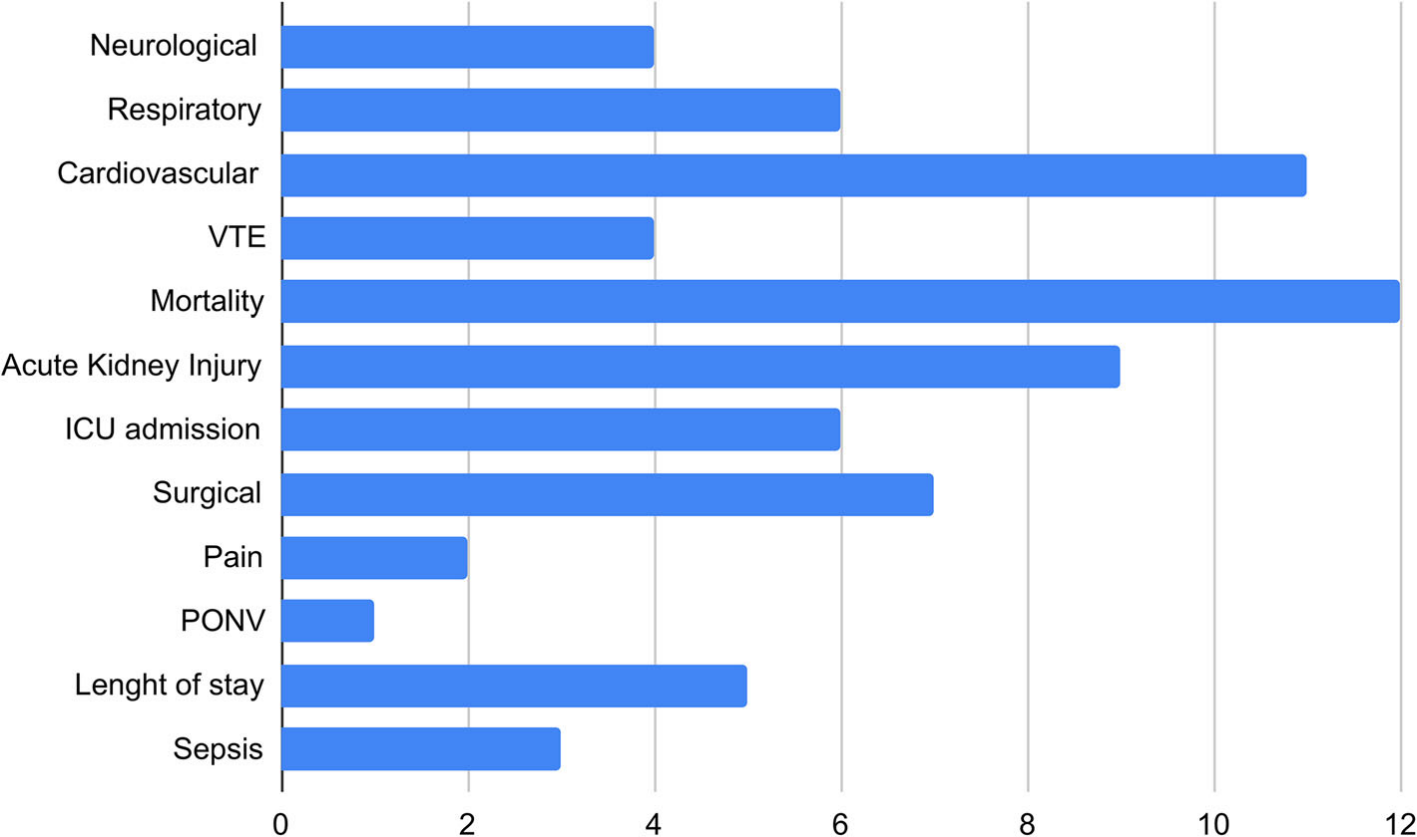
Main outcomes (preoperative/intraoperative) considered in our analysis

The use of these new technologies to analyze perioperative complications has been tested in almost all types of surgery (general, cardiac, orthopedic, neurosurgical, vascular). Variables and predictors were properly listed and described in all the articles. ML methods were used mainly to predict the following outcomes: mortality (*n* = 12), cardiovascular complications (*n* = 11), acute kidney injury (AKI; *n* = 9), surgical complications (*n* = 7), intensive care unit admission (ICU; *n* = 6), respiratory complications (*n* = 6), length of stay (*n* = 5), venous thromboembolism (VTE; *n* = 4), neurological complications (*n* = 4), sepsis (*n* =3), pain (*n* = 2), and post-operative nausea and vomiting (PONV; *n* = 1) (Fig. 4).

As stated before, most of studies considered preoperative variables, like demographic, medical history, clinical and laboratory values evaluation, to calculate perioperative risk. Conversely, several studies evaluated intraoperative variables, as electroencephalography (EEG) pattern [34], or intraoperative vital signs [13, 15, 22, 24, 46, 47], for a real-time prediction of overly deep sedation, post-induction and intraoperative hypotension, hypoxemia, and intraoperative bradycardia.

**Fig. 5:**
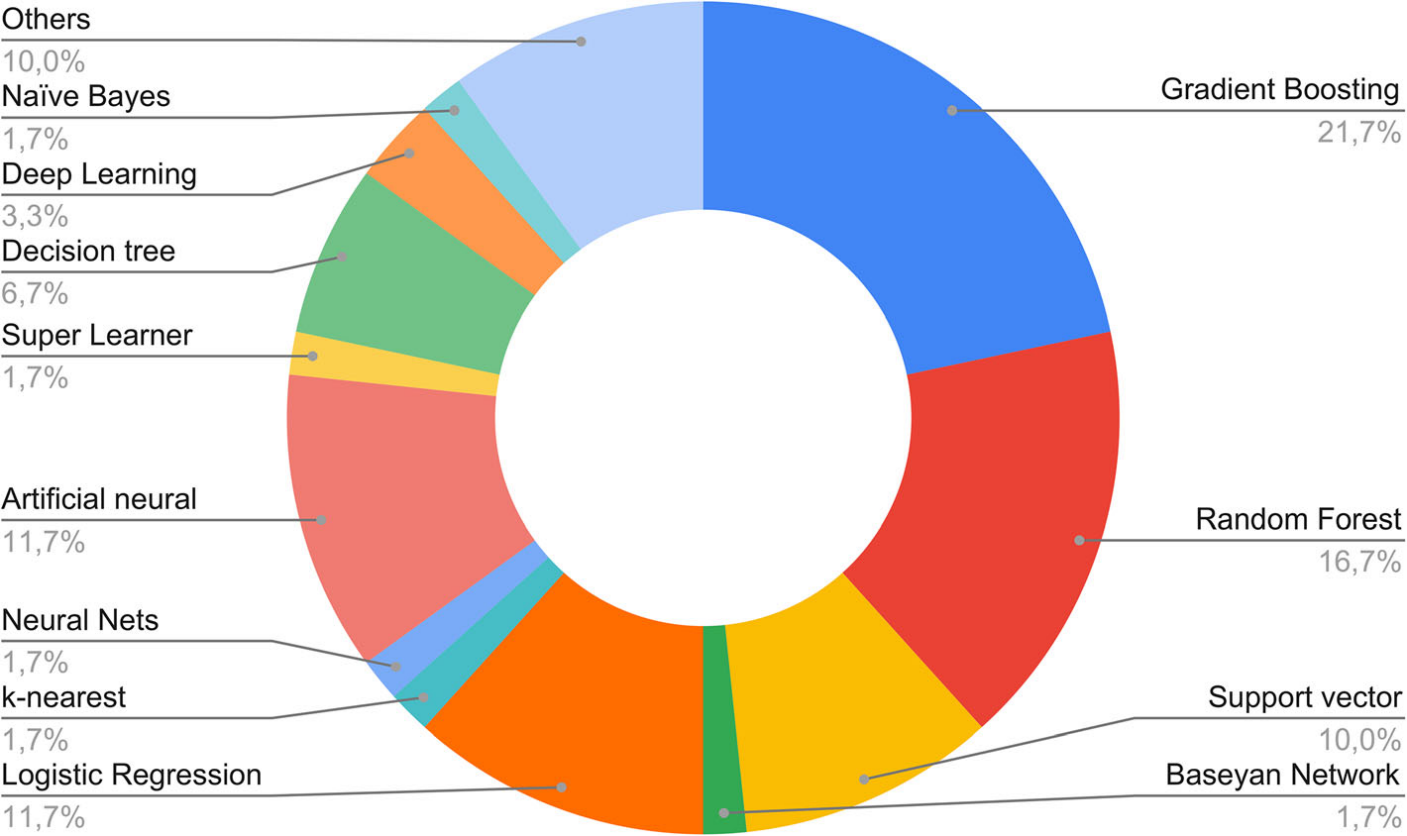
Presentation of the main types of machine learning methods used in the analysis of our studies

Supervised models were used in most of cases (Fig. 5). The most frequently used algorithms were gradient boosting (*n* = 13), random forest (*n* = 10), logistic regression (LR; *n* = 7), artificial neural networks (ANNs; *n* = 6), and support vector machines (SVM; *n* = 6). Deep learning, decision trees, and Naïve Bayes were other models commonly applied in the included manuscripts.

In the totality of reviewed papers, ML algorithms proved to be effective in outcome prediction. Half of the selected studies compared different types of ML to identify the best performing method. Gradient boosting and random forest were found to be the models with the highest accuracy, achieving an area under the curve (AUC) greater than 0.90 in most of cases. Moreover, a few studies compared automatically obtained algorithms to conventional scores, revealing the outperformance of ML models [25].

## Discussion

The number of manuscripts regarding ML implementation in health care settings is steadily increasing over the last few years, as clearly suggested by a recently published review on AI utility to provide decision support to clinicians in ICU setting [49, 50].

In fact, the availability of electronic health records, and the diffusion of Big Data systems have enabled new possibilities in data collection and storage. The interpretation of this amount of data with traditional methods could not only be extremely complicated, but even reductive. In this regard, the advent of AI-based technologies has opened up new perspectives, providing a different form of research [51].

Anesthesia and assessment of perioperative risk appear to be excellent fields to develop and apply ML systems, as reported in literature [52, 53], and confirmed by our research. The identification of modifiable risk factors and the subsequent optimization of the preoperative phase appear to be a crucial factor to decrease the incidence of post-operative complications [54]. Furthermore, risk stratification allows the acquisition of an adequate informed consent and an accurate anesthesiologic planning, tailored to each patient. ML systems are well suitable for this context, where the possibility to collect a large number of data and the choice of the variable that is selected by the model itself, allows the discovery of new factors and a different interpretation of already known items. Thus, the availability of interpretations and predictions in real time could allow to enter a new era of anesthesia.

From a practical point of view, the method starts with multi-source data extrapolated and collected; subsequently, they are placed in ML systems able to return interpretative and predictive models, providing suitable tools for daily technologies with validated scores. Among conventional scores, the one used more frequently for comparison is the ASA-PS Classification System that has been in use for over 60 years. Comparing existing scores with new models is an essential step to understand whether this investment of time and resources could finally improve the perioperative risk stratification.

Moreover, in addition to the risk of post-operative complications, ML would also be able to answer more complex questions and create models capable of providing early predictions of adverse events, thus enabling a perioperative optimization.

The results that emerge from this systematic analysis are promising. In studies that compared ML models with traditional scores, most confirmed their outperformance. In particular, the use of AI-based technologies provided excellent results regarding events of great interest in the field of Anesthesia, as post-induction hypotension and post-intubation hypoxia [13], or the risk of AKI or delirium after surgery [19, 27, 55].

**Fig. 6:**
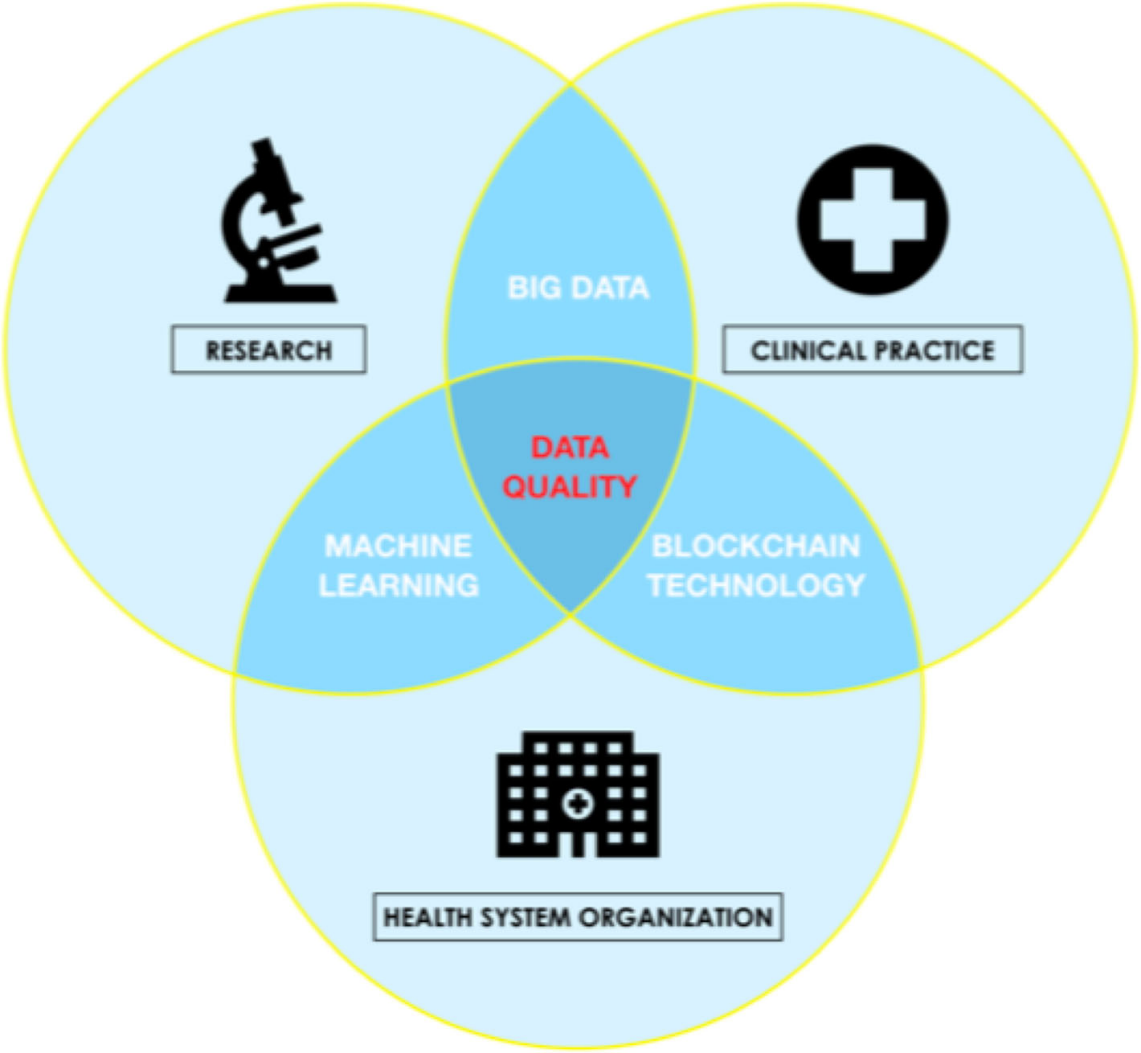
Importance of acquisition of data quality for application of AI in different fields such as research, clinical practice, and health system organization

Finally, it is interesting to underline that not only clinical outcomes are relevant, but also administrative ones, as length of hospital stay, or need for recovery in intensive care settings, that may have a great relapse into hospital logistics and in economic strategies (Fig. 6). A systematic use of AI might allow the achievement of innovative results in other fields as well, such as scientific research and health organization, especially when associated with other data management technologies such as Big Data and Blockchain.

Among several ML algorithms currently applied, Gradient boosting and random forest were found to be the models with the best performance and the highest accuracy, achieving an area under the curve (AUC) greater than 0.90 (Ref #2, comm #3). Still, it is not possible to make a uniform evaluation and draw conclusions about the best algorithm for predictive models of perioperative complications, because of the heterogeneity of settings and the difference in the algorithms evaluated. The lack of uniformity of the included studies prevented us from performing a meta-analysis using univariate and multivariate random effect models (Ref #2, comm #3). Moreover, the models in most of the studies lack an external validation.

Further, even if we practically use AUC as an evaluation criterion, we acknowledge its limits in the setting of AI, especially in case of unbalanced dataset. Note that other criteria can also be used to evaluate ML models, such as model relevance, efficiency, and interpretability [56]. However, to achieve high-quality and high-quantity data sets, it is of paramount importance the screening of each step of the process, from data collection to ML model selection and its algorithm

(Rev #2, comm #3, comm #4).

Despite their growing diffusion, the use of these technologies in perioperative medicine is raising limitations and challenges. Along with technological progress, data quality will inevitably become increasingly important. A viable choice could be blockchain technology, to ensure adequate quality and enable secure data sharing. Its implementation could allow the safe management of large files and consequently the approval of algorithms that are progressively developed [57].

Furthermore, as recently reported for ICU-setting [50], despite the potential role of AI to improve clinical outcomes, the vast majority of developed models remain within the testing and prototyping environment. A uniform and structured approach could enable the implementation and safe delivery of AI technologies in ICU and overall, in health care settings.

Finally, the creation of predictive scores should guarantee precise rules. Unfortunately, these technologies are so innovative that the evaluation of their performance is not always so linear. Therefore, a new version of the TRIPOD statement specific for AI/ML systems (TRIPOD-ML) is currently under development. It will focus on the introduction of ML prediction algorithms to establish methodological and reporting standards for ML studies in health care [58].

Technologies are becoming more and more present in health-care settings. Both clinical and organizational decision-making processes can take advantage of these technologies. Nevertheless, high-quality studies are needed to demonstrate the real impact of ML in this context.

Our research group is starting a study that aims to validate a safe discharge score from the PACU (post-anesthesia care unit) using AI techniques; the score will no longer be generic, but based on the local clinical reality and on the specific population. Similarly, we are working on the application of AI algorithms in OR (operating room) management settings, developing a prospective trial “Bloc-op” (NCT 05106621), in collaboration with the engineering department, to optimize OR organization and resources allocation. We believe that multidisciplinary collaboration is essential to integrate AI technologies into routine clinical practice, thus leading to a great improvement in the quality of care.

We proposed that AI should become an essential technical and non-technical skill for the future anesthesiologists. In order to achieve this goal, a primary focus should be the education and training of physicians and researchers, who need to be adequately prepared on the uses and limitations of AI based technologies (Rev #2, comm #4).

## Conclusions

This systematic review shows the potential role of ML in perioperative medicine, and particularly in the creation of models for the prediction of perioperative risk. Our results are encouraging.

Undoubtedly, the exploitation of a large amount of data is possible solely thanks to the application of AI. ML algorithms offer increasingly precise solutions in terms of optimization of the perioperative risk. A personalized risk/benefit analysis can result in an accurate prediction in terms of length of hospital stay and ICU recovery, thus positively influencing patient management and health costs.

Further research is needed to develop a framework standardizing AI evaluation measures, and this will be possible with interdisciplinary approaches, allowing to constantly improve high-quality care.

## Data Availability

Not applicable

## Abbreviations

ML: Machine learning
PRISMA: Preferred Reporting Items for Systematic Reviews and Meta-analyses
ICU: Intensive care unit
TRIPOD: Transparent Reporting of a Multivariable Prediction Model for Individual Prognosis or Diagnosis
AKI: Acute kidney injury
LR: Logistic regression
ANNs: Artificial neural networks
SVM: Support vector machines
ASA-PS: American Society of Anaesthesiologists Physical Status
EuroSCORE: European system for cardiac operative risk evaluation
AI: Artificial intelligence
EEG: Electroencephalography
AUC: Area under the curve
TRIPOD-ML: Transparent Reporting of a Multivariable Prediction Model for Individual Prognosis or Diagnosis-Machine Learning
PACU: Post-anesthesia care unit
OR: Operating room
